# Attitudes towards the COVID-19 Vaccine and Willingness to Get Vaccinated among Healthcare Workers in French Guiana: The Influence of Geographical Origin

**DOI:** 10.3390/vaccines9060682

**Published:** 2021-06-21

**Authors:** Nicolas Vignier, Kepha Brureau, Sybille Granier, Jacques Breton, Céline Michaud, Mélanie Gaillet, Camille Agostini, Mathilde Ballet, Mathieu Nacher, Audrey Valdes, Philippe Abboud, Antoine Adenis, Félix Djossou, Loïc Epelboin, Maylis Douine

**Affiliations:** 1 Centre d’Investigation Clinique Antilles Guyane, CIC Inserm 1424, DRISP, Centre Hospitalier de Cayenne, Av des Flamboyants, 97300 Cayenne, France; k97.brureau@gmail.com (K.B.); graniersibylle@gmail.com (S.G.); mathieu.nacher66@gmail.com (M.N.); antoine.adenis@ch-cayenne.fr (A.A.); maylis.douine@ch-cayenne.fr (M.D.); 2 Institut Pierre Louis d’Épidémiologie et de Santé Publique (IPLESP), Department of Social Epidemiology, Sorbonne Université, 75012 Paris, France; 3 UFR SMBH, Faculté de Médecine, Université Sorbonne Paris Nord, 97300 Bobigny, France; 4 Département Universitaire de Médecine Générale, Université des Antilles, 97145 Pointe-à-Pitre, France; 5 Département Universitaire de Médecine Générale Montpellier-Nîmes, Université de Montpellier, 34090 Montpellier, France; 6 Union Régionale des Professions de Santé—Médecins Libéraux de Guyane, 97300 Cayenne, France; jacques.14@icloud.com; 7 Centres Délocalisés de Prévention et de Soins, Centre Hospitalier de Cayenne Andrée Rosemon, 97300 Cayenne, France; celine.michaud@ch-cayenne.fr (C.M.); melanie.gaillet@ch-cayenne.fr (M.G.); 8 Centre Hospitalier Ouest Guyanais, 97320 Saint Laurent du Maroni, France; c.agostini@ch-ouestguyane.fr; 9 Agence Régionale de la Santé de Guyane, 97300 Cayenne, France; mathilde.ballet@ars.sante.fr; 10 Campus de Troubiran, DFR Santé, Université de Guyane, 97337 Cayenne, France; felix.djossou@ch-cayenne.fr (F.D.); epelboincrh@hotmail.fr (L.E.); 11 Hygiene Department, Centre Hospitalier de Cayenne Andrée Rosemon, 97306 Cayenne, France; audrey.valdes@ch-cayenne.fr; 12 Unité des Maladies Infectieuses et Tropicales, Centre Hospitalier de Cayenne Andrée Rosemon, 97306 Cayenne, France; philippe.abboud@ch-cayenne.fr

**Keywords:** COVID-19 vaccines, health personnel, vaccine hesitancy, willingness to get vaccinated, French Guiana, South America

## Abstract

*Background:* In the context of the global COVID-19 pandemic and the expansion of the more transmissible 20J/501Y.V3 (Gamma) variant of concern (VOC), mRNA vaccines have been made available in French Guiana, an overseas French territory in South America, from mid-January 2021. This study aimed to estimate the willingness to be vaccinated and the socio-demographic and motivational correlates among Health Care Workers (HCWs) in French Guiana. *Methods:* A cross-sectional survey was conducted from January 22 to March 26, 2021 among a sample of HCWs in French Guiana. They were asked about their willingness to get vaccinated against COVID-19 and vaccine hesitancy, vaccine uptake and vaccines attitudes. Factors associated with willingness to get vaccinated have been analyzed with ordinal logistic regression, using Stata software. *Results:* A total of 579 HCWs were interviewed, including 220 physicians and 200 nurses most often working in hospital (54%) or in the liberal sector (22%). Overall, 65.6% of respondents reported that they were willing or had already been vaccinated against COVID-19, while 24.3% of respondents reported that they did not want to get vaccinated against COVID-19 and 11.2% were unsure. HCWs were more willing to get vaccine if they were older, were worried about COVID-19 and were confident in the management of epidemic. Conversely, participants were less likely to have been vaccinated or willing to if they were nurses or of another non-medical profession, born in French Guiana, feared adverse effects, or if they did not trust pharmaceutical companies and management of the epidemic by authorities. *Conclusion:* Negative attitudes towards vaccines are a major public health concern among HCWs in French Guiana when considering the current active epidemic with Gamma VOC. General vaccine hesitancy and concerns about future side effects in particular represent important barriers. Low confidence in government and science are significant in COVID-19 vaccine refusal among non-medical staffs. Public health messaging with information on vaccine safety should be tailored to address these concerns. The specific challenges of HCWs from French Guiana must be taken into account.

## 1. Introduction

Since its emergence in late 2019, severe acute respiratory syndrome coronavirus-2 (SARS-CoV-2) has spread worldwide with huge health and socio-economic consequences. With a basic reproductive number of 3 in the absence of prevention measures [1], a high level of herd immunity seems necessary to improve the situation [2]. COVID-19 vaccines were therefore an eagerly awaited component of the pandemic response. When the preliminary results on the efficacy of the vaccine BNT162b were published in December 2020, Europe, including France, implemented free vaccination campaigns [3]. As recommended by the World Health Organization (WHO), healthcare workers (HCWs) were considered a priority group for COVID-19 vaccination [4]. However, in addition to the supply difficulties and the logistical challenges of scaling-up vaccination, the issue of vaccine-hesitancy in the population, and in particular among HCWs, became prominent [5].

The WHO has named vaccine hesitancy as one of the top ten threats to global health in 2019 [6]. Vaccine hesitancy refers to a delay in acceptance or refusal of vaccines despite availability of vaccination services and is determined by complacency, convenience and confidence, as well as by individual and contextual factors [7]. France was already one of the countries with the strongest suspicion towards vaccines worldwide [8,9]. This may be related to previous health scandals such as the contaminated blood affair, or to the dissemination of misinformation such as the alleged link between the hepatitis B vaccine and multiple sclerosis, but also for many other reasons [10,11]. More recently, the massive orders and non-use of anti-influenza A(H1-N1) vaccine in 2009 was seen as indicative of a lack of transparency between politicians and the pharmaceutical industry [12]. The rapid delays in the marketing authorization of COVID-19 vaccines may have amplified this feeling of mistrust. Despite the scientific and professional information available to them, HCWs are subject to vaccine hesitancy, just like the rest of the population [8,13,14]. Vaccine hesitancy-associated factors are mainly represented by perceived susceptibility, lack of information, fear of side-effects, individual belief, awareness of health behaviors, and social context [14,15]. Social media use plays a role in vaccine hesitancy [16,17].

Previous studies have examined predictors of intent to get vaccinated against COVID-19 in the general population, and there have been few studies since their availability [18,19,20]. Reasons for unwillingness to receive the COVID-19 vaccination concerned its novelty, safety, and potential side effects [5]. Socio-demographic predictors of uncertainty and unwillingness to vaccinate include female gender, low socio-economic status, not getting flu vaccine last year, poor adherence to COVID-19 government guidelines, concerns about the unforeseen early and late side-effects of vaccines and general mistrust in the benefits and safety of vaccines [20,21]. Additionally, among a representative sample of the French general population, similar results were found [22].

Negative attitudes towards vaccines and unwillingness to receive vaccinations are major barriers in the COVID-19 pandemic control, hampering the goal of herd immunity. This is of particular concern among HCWs, given their role as a potential source of contamination of patients, the weight of absenteeism on the stability of the healthcare system and their public health mission to promote vaccination [15]. Among HCWs, physicians have been reported more willing to get vaccinated against COVID-19 than other HCWs, while the highest anti-vaccination attitudes were observed among nurses [23].

French Guiana (FG) is a French overseas territory in South America, located in the Amazon region. The population has a low density (3.2 inhabitants per km^2^), is young (77% of the population is under 45), multicultural (Creole, Bushinengue, Brazilian, Haitian, Amerindian, Surinamese, Guyanese, European, Dominican, etc.), and often poor (half of the population lives below the poverty line) and illiterate (48.7% of the population has an educational level of primary school or less) [Census 2017 data, Insee]. In this context, COVID-19 dynamics were very different from Europe [22]. In the Amazonian region in general the growth speed was generally slower than in Europe or the USA, or Southern Brazil [24]. FG faced the first wave two months after Europe [25]. The infection attack rate of SARS-CoV2 was, however, one of the highest in France, but the number of hospitalizations and deaths was lower. This may be linked to the youth of the FG population and/or to the 2 month delay with mainland France with improvements in patient care (anticoagulation, steroid and ventilation) [26].

Although the combination of strict interventions including curfews and localized lockdowns brought the situation under control relatively quickly [26], the socio-economic consequences were very significant in this precarious department [25,27]. Indeed, the COVID-19 epidemic has been accompanied by the cessation of many formal and informal activities and a significant increase in food insecurity. On the health front, the chronic shortage of health professionals has strained the health system, thus requiring reinforcements from the national health reserve. A concomitant dengue epidemic also contributed to the exhaustion of professionals [28]. Moreover, the proximity with Brazil, where the pandemic is raging, promoting the emergence of more contagious variants, exposes it to the arrival of a third intense wave with the 20J/501Y.V3 (P.1) variant of concern (VOC) [29,30]. It is therefore essential to protect health care workers in this isolated territory, 8000 km from Europe, in order to maintain an efficient health care system, at a time when French hospitals are overwhelmed, and reinforcements would be difficult to obtain. In July 2020, the overall seroprevalence in FG was estimated at 15.4% (95%CI 9.3–24.4) [31]. In January 2021, when the vaccine became available in FG, about 12,000 COVID-19 cases had been notified among a population of 300,000 inhabitants. Reaching herd immunity thus requires a strong participation to vaccination. At the time of writing, the epidemic is accelerating alarmingly with an incidence greater than 380/100,000 and a predominance of the Gamma VOC (>80%).

French Guiana started vaccinating healthcare workers (HCWs) over 50 years of age, nursing home residents and people at high risk of the complications of COVID-19 with the COVID-19 BNT162b mRNA (Comirnaty^®^, Pfizer^®^-BioNTech^®^) vaccine, chosen because of its supposedly better efficacy on the Gamma VOC than that of AstraZeneca-mid-January 2021 [32]. From mid-February vaccine indications were extended to all HCWs. Very quickly, negatives attitudes were observed. At the end of February 2021, only 679 out of 4151 HCWs (16.4%) and 3800 of the 294,071 inhabitants (1.3%) were vaccinated against COVID-19. A better understanding of the attitudes and vaccination intentions of HCWs in this particular territory is thus necessary to adjust the campaign and the information provided in order to increase vaccine adhesion [33].

The present study aimed to assess attitudes to the COVID-19 vaccine among HCWs in French Guiana during the first two months of the vaccination campaign.

## 2. Methods

### 2.1. Study Design

We led a descriptive cross-sectional survey from 22 January to 26 March 2021 among HCWs in French Guiana.

### 2.2. Study Population

All HCWs working in FG at the time of the survey, in private or public practice and agreeing to participate were eligible. Vaccination was accessible to all HCWs from mid-February through vaccination centers within hospitals. Thus the whole study population was directly concerned by the concomitant vaccine campaign.

### 2.3. Sampling and Procedure

The study was conducted using an auto-administered semi-structured online questionnaire. An online platform (https://www.wepi.org/, Epiconcept^®^ (accessed on 18 June 2021) with certified server to host personal health data was used to conduct the survey. All possible and available diffusion lists were used to reach all HCWs across French Guiana. HCWs were approached by mail from the heads of the 3 main hospitals, of the 17 public prevention and care centers (PPCC) in isolated villages, of the regional union of liberal doctors and nurses, by the Regional Health Agency (weekly letter), WhatsApp groups and professional mailing lists and were recalled by phone or physically by medical residents. A paper, anonymous version of the questionnaire was also made available in PPCC for those having internet access difficulties. A reminder displaying a QR code was on the desktop of all the computers of the main hospital in Cayenne and by service executives.

### 2.4. Data Collection

Data were obtained directly from participants. The questionnaire was based on those used in mainland France in order to compare the results [34]. It contained socio-demographic data, a representation of vaccines in general and of the COVID-19 vaccine in particular, as well as willingness to get vaccinated and its associated factors. Additional data were added, such as the origin of health professional, activity and mode of practice, as well as qualitative precision about obstacles and levers to vaccination in regard to FG specificities.

### 2.5. Statistical Analysis

The data were described using standard tests with Stata© 15.1 software (StataCorp, College Station, TX, USA). The primary endpoint was willingness to get vaccinated (Already Vaccinated or Likely/Unsure/Unlikely or Sure not to get Vaccinated). Qualitative variables were described as percentages and quantitative variables, as median and interquartile range. Chi-squared or Fisher’s exact tests were used for cross-tabulations of qualitative variables, as appropriate.

Univariate and multivariate analyses were performed using ordinal logistic regression models. Analyses examined the impact of socio-demographic and occupational data, knowledge, attitudes, and representations of vaccines in general on the willingness to get vaccinated against COVID-19. Associations were expressed using odd ratios and 95% Confidence Intervals. In our final models, we included all statistically significant confounders using a threshold of 0.20 for the *p*-value. When several associated factors explored the same dimension (attitude towards vaccination in general for example), the choice of a single variable was made.

### 2.6. Ethics and Regulation

Data were collected in a strictly anonymous manner with the participants’ consent collected online on the accredited website https://www.wepi.org/ (accessed on 18 June 2021). The collection of data has been subject to the individual information of participants, a privacy impact analysis and the study online deposit on the French Health Data Hub platform in accordance with the French and European General Data Protection Regulations (RGPD). No ethical approval was required in accordance with the Jardé law. Participants were informed in accordance with Article 13 of the RGPD of their right not to participate and to request access to their answers.

## 3. Results

During the two months of study, a total of 579 HCWs answered the questionnaire, an estimated response rate of 13.9% (579/4151).

### 3.1. Characteristics of Participants and Their History and Concerns about COVID-19

Characteristics of the participants are presented in Table 1. Most participants were female (67.9%), born in mainland France (59.9%) or from French Guiana (19.9%), were in contact with patients (84.4% in total, among which 38.0% of physicians and 34.5% of nurses), 14.3% participants reported health conditions at risk of severe COVID-19 and 19.0% reported personal history of COVID-19, most often at the hospital or in remote area care centers (Table 2). More than half of HCWs had no or little confidence in the impact of case management and they were worried about the COVID-19 epidemic; most of them had faced severe COVID-19 cases in practice.

### 3.2. Attitude towards Vaccination in General

In total, 90.9% of the respondents were totally or rather favorable to the vaccination in general. Some (30.1%) were unfavorable to certain vaccinations (Table 3). Distrustful attitudes towards vaccination were more frequent amongst nurses and other non-medical HCWs, among the youngest HCWs (11.9% under 50 s vs. 3.8% over 50 s unfavorable to vaccinations, *p* = 0.002), HCWs from French Guiana or the West Indies (28.8% vs. 3.7% for others, *p* < 0.001), HCWs considering themselves not informed about vaccination (22.9% vs. 6.3%, *p* < 0.001) and those who did not trust information from the health authorities (33.6% vs. 2.4%, *p* < 0.001).

Although the majority reported being up to date with their vaccinations, less than a third of respondents had taken the flu shot in the past two years, in connection with a lack of confidence in its efficacy and tolerance. A third of non-medical staff said they lacked information about vaccinations.

### 3.3. Attitude towards COVID-19 Vaccine

Overall, 64.4% of respondents reported that they were willing to get, or had already been, vaccinated against COVID-19, while 24.4% of respondents reported that they did not want to get vaccinated against COVID-19 and 11.2% were unsure. In multivariate analysis (Table 4), after controlling for all other risk factors, gender, age, country of birth, profession, worry about COVID-19, confidence in the management of the epidemic, attitudes towards vaccination in general and confidence in pharmaceutical companies remained significantly associated with vaccine willingness. Participants were less likely to have been vaccinated or willing to get vaccinated if they were born in French Guiana or the French West Indies, if they were nurses or another non-medical profession, or if they did not trust pharmaceutical companies and the management of the epidemic by authorities. Female gender was a predictor of both COVID-19 vaccine uncertainty and refusal but was no longer significant once attitude towards vaccinations in general was taken into account.

Conversely, HCWs were more willing to get vaccinated if they were older, were worried about COVID-19 and were confident in the management of epidemic.

The other determinants of a favorable attitude to vaccination in general (level of information, authorities’ confidence, altruistic conception of vaccination, perception and practice of influenza vaccination last year) were also strongly correlated with COVID-19 vaccination willingness but were not integrated into the multivariate model, given their collinearity (Table 4).

The two main motivations for vaccination among those willing to get vaccinated were the efficacy against severe COVID-19 (60.6%) and limitation of virus transmission (59.0%). They differed from the hesitant participants who expect more follow-up (66.0%) and a guarantee of effectiveness and absence of adverse effects (42.7%).

The most important determinants of uncertainty and reluctance to vaccinate against COVID-19 were intermediate to high doubt about the benefits of the vaccine and concern about possible side effects (Table 5).

Attitudes towards the COVID-19 vaccines were more negative compared to attitudes towards vaccines in general (65.6% willing to get vaccinated against COVID-19 vs. 90.9% very or rather favorable to vaccinations) and 23% considered that COVID-19 vaccine has more side effects than other vaccines.

HCWs unfavorable to a specific vaccine were less willing to get vaccinated against COVID-19, especially for those opposed to vaccinations against hepatitis B (*p* = 0.04), human papillomavirus (*p* < 0.001), influenza (*p* < 0.001) and shingles (*p* = 0.02).

The attitudes towards the COVID-19 and influenza vaccines were correlated since 90.7% of those vaccinated against influenza in 2019–2020 plan to get vaccinated against the COVID-19.

Conversely, the better informed the caregivers were about vaccines, the more they were willing to get COVID-19 vaccine (*p* < 0.001). This observation differed according to the sources of information: HCWs who use social networks as a source of information were less willing to get vaccinated against COVID-19 (40.3% vs. 67.9%, *p* < 0.001).

Willingness to get vaccinated against COVID-19 for oneself was strongly correlated with vaccine recommendation for relatives and for patients: 93.0% of HCWs vaccinated or willing to get vaccinated would be ready to recommend the vaccine to their relatives and 91.2% to their patients. These percentages were 21.5% and 35.3% for the unsure. However, 11.0% of caregivers opposed to vaccines were still ready to recommend it to their relatives and 20.4% to some of their patients (*p* < 0.001).

In an open question about the challenges for vaccination considering the specificities of French Guiana, HCWs answered regarding the need for understandable information (24.9%), the frequency of comorbidities as diabetes, obesity and hypertension (12.3%). The barriers that were put forward were the lack of time since the introduction of vaccines (15.9%), the cultural and borders issues (14.0%), logistical issues (12.6%), the poorly known efficacy (10.7%) and adverse events (6.9%), the unknown efficacy on Gamma VOC (8.1%), precariousness (7.4%) and the inadequacy of the health system (2.9%).

## 4. Discussion

This first study on COVID-19 vaccine hesitancy among HCWs at the beginning of the vaccination campaign in French Guiana showed that 64% were ready to get vaccinated with a gradient according to profession, age, gender, origin and attitudes towards vaccination.

### 4.1. A Vaccination Intention of HCWs Similar to Mainland France but Still Lower Than in the Rest of Europe

The vaccination intention rate in HCWs in French Guiana in early 2021 is similar to that observed in studies among HCWs in mainland France in October 2020 (68% [34]) and October–November 2020 (75% [35]), and to other French-spoken countries (76% in Belgium). Previous studies had already shown that vaccine hesitancy could exist among French family physicians and other HCWs [35,36]. In the two studies cited above, the factors associated with the vaccination intention of HCWs in France were older age, male gender, profession (physicians and nurses supervisors), fear of COVID-19, taking the influenza vaccine, trust in science and the Ministry of Health and not being worried about COVID-19 vaccine safety [34,35].

Distrust of vaccines is a well-known phenomenon in France, a country which was identified as the most hesitant in a 67-country survey [8]. French hesitancy against the COVID-19 vaccine was already measurable in the general population at the height of the first wave in March 2020 [37]. COVID-19 vaccination was lower than in other European countries [38,39]. The COVID-19 vaccine refusal was 29.4% (95% CI 28.6–30.2) of the French working-age population and was significantly associated with female gender, age, lower educational level, poor compliance with recommended vaccinations in the past, no chronic conditions and lower perceived severity of COVID-19 [9,22].

International studies shed light on the determinants of the vaccination intention of HCWs. In a recent large study among health-care workers in England (SIREN) where 89% of 23,324 participants were vaccinated, significantly lower COVID-19 coverage was associated with previous infection, female gender, age, ethnicity, job role, and Index of Multiple Deprivation [21]. Similar results had been reported by other studies [5,40,41,42]. COVID-19 vaccine intention was also reported to be lower among nurses than among physicians [5].

### 4.2. Higher Vaccine Hesitancy among Women and Nurses/Caregivers

In our study, as in those conducted in mainland France or other countries such as Israel [5], women and nurses/caregivers were more reluctant to get vaccinated against COVID-19 than men and physicians and midwives [34]. A study in Hong Kong estimated nurses’ intention to receive the COVID-19 vaccine (63%) and associated vaccine hesitancy factors (more confidence, less complacency and more collective responsibility) and greater work stress [43]. In another study, a major concern of nurses about the COVID-19 vaccine was its efficacy and safety [23]. In addition, there is a link between poor perception of management or suffering at work and refusal of the influenza vaccine, the vaccine being a pretext (rather than a reason) for expressing dissonance with working conditions deemed inadequate [44]. Caregivers’ willingness to be vaccinated would thus be influenced by perceptions of institutional and structural discrimination [19].

### 4.3. Specificities in French Guiana

A striking observation was that caregivers born in FG are less favorable to vaccination in general and against COVID-19 in particular than other HCWs. The meaning of vaccination hesitance depends on the specific context. The population of French Guiana is multicultural with French Creoles from French Guiana and French West Indies, French from mainland France, Maroons, Amerindians, Hmongs, migrants (recent or long established) from Brazil, China, Suriname, Haiti, Guyana and Dominican Republic. The HCWs participating in the study were born mainly in mainland France, the French Guiana and the French West Indies. The other caregivers were from 42 different countries. This great cultural diversity, which is not the same within the HCWs and within the population receiving care, makes French Guiana probably one of the territories with the greatest diversity of caregiver-patient cultural interactions. A study, not yet published, was conducted about vaccination intentions in the global population by the Pasteur Institute among 1348 inhabitants from French Guiana [45]. It showed that less than one in two planned to get vaccinated and that reluctance to vaccines was linked to the fear of ineffectiveness in the context of the circulation of new VOC, the fear of side effects, the fear that electronic chips are present in vaccines to control individuals, and the preference for traditional remedies. As of 15 May 2021, only 4.3% of the population is fully vaccinated against COVID-19 in French Guiana (vs. 12.6% in mainland France), despite the reactivity of the health authorities who extend the indication for vaccination to all people over the age of 16 as soon as the target population no longer comes to the vaccination center [46]. The interpretation of the significantly greater distrust of health professionals born in French Guiana and the French West Indies underlines a general ambivalence towards mainland France, with negative attitudes (feeling of neglect or being France’s “guinea pigs”) that are grounded in the history of past centuries but often amplified by local politicians who stroke this distrust for political gains.

### 4.4. Impact of These Findings on Health Policy in French Guiana

Health care professionals are a priority group for vaccination against COVID-19 everywhere. In French Guiana, this is even more important: being several thousand kilometers from mainland France and suffering from a chronic shortage of health care personnel, the increased workload associated with the COVID-19 pandemic is a real problem. The protection of caregivers is therefore essential.

At the end of April, there were 1945/4151 HCWs (46.7%) vaccinated in French Guiana (first dose), of whom 1253 had received their second dose (Data from the regional Health Agency of French Guiana).

One of the main obstacles being the novelty of this vaccine, we can expect that coverage will continue to improve in the coming months as knowledge of efficacy and adverse events increases. It is all the more important that HCWs influence and promote immunization in the general population. Their own opinions and actions (i.e., being vaccinated) necessarily influence those of their patients.

### 4.5. Levers to Improve Vaccine Coverage in French Guiana

According to our results, information about vaccines is one of the cornerstones of HCWs’ vaccine adherence. Vaccine safety communication to increase HCWs trust should be reinforced. In addition, social networks play an important role in the dissemination of false messages about vaccination in the general population and among HCWs [17]. Contradictory communication from authorities, experts and opinion leaders during the crisis increased the use of social networks. It is therefore crucial to provide updated, clear and independent information to all HCWs regularly, considering the fast evolution of knowledge. Scientific information and recommendations evolve very quickly, and the caregivers, overwhelmed by the care, do not have the ability to read everything

Nonprofit organizations and health authorities are also working to provide information to the population in different languages and using different tools adapted to the different communities. This will help HCWs provide information and discuss the vaccine with patients, given the great cultural diversity of the HCWs and the population. However, perceived lack of information by health professionals is still somewhat surprising given the abundance of official information or available publications. The reported “lack of information” may have reflected more negative attitudes and distrust of information from authorities and science, attitudes that breed conspiracy theories with an overseas French territories’ colonial twist. Therefore, how vaccination is framed should matter: if it is perceived as the passive implementation of the mainland’s decrees, vaccination will raise some resistance, but if it is framed as the effort of French Guiana to protect its own, perhaps attitudes may change.

### 4.6. Strengths and Limitations

There were 544 physicians and 1853 nurses in French Guiana in 2019 [47]. Thus, it is 40.4% of physicians and 10.8% of nurses in French Guiana who responded to the survey. Although not perfect, the representation of doctors and nurses is therefore satisfactory. However, other paramedic staff are poorly represented. Personalized reminders by medical assistants tried to reduce this selection bias. Overrepresentation of HCWs willing to get vaccinated and less against the system is possible, given the sampling method. During physical and phone reminders, some people declared that they were unwilling to participate because they perceived the study as some kind of control, an attitude that may indicate a greater reluctance to follow recommendations. In this context, local opinion leaders may have a role in convincing health-workers and the population about the benefits of the vaccine.

## 5. Conclusions

A significant number of HCWs in French Guiana are still hesitant about the COVID-19 vaccine as the epidemic intensifies with Gamma VOC, more often among paramedics and HCWs born in French Guiana. Safety, effectiveness, and speed of development were noted as the most common concerns regarding COVID-19 vaccination. Low confidence in government and science were associated with COVID-19 vaccine refusal among non-medical staffs. This mistrust and specific obstacles must be addressed on a personal and global scale by involving supervisors, health authorities and opinions leaders. Public health messaging with information on vaccine safety should be tailored to address these concerns. The specific challenges of HCWs from French Guiana must be taken into account.

## Figures and Tables

**Table 1:** Characteristics of participants.

		Number of Respondents	*n*	%
Total		579	579	
Gender		579		
	Women		393	67.88
	Men		186	32.12
Age (years)		579		
	18–34		187	32.30
	35–49		198	34.20
	50–64		152	26.25
	65+		42	7.25
Country of birth		579		
	France (mainland)		347	59.93
	French Guiana		115	19.86
	Others French overseas territories		18	3.11
	Brazil		10	1.73
	Guyana		1	0.17
	Surinam		3	0.52
	South America (others)		9	1.55
	Africa		39	6.74
	European Union		18	3.11
	Others		10	1.73
Language spoken at home		579		
	French		537	92.75
	Others		42	7.25
Year of arrival if born outside French Guiana		464		
	Median, IQR		2013	[2002–2019]
Profession		579		
	Physician		220	38.00
	Midwife		24	4.15
	Nurses		200	34.54
	Health-care assistant		9	1.55
	Nurses supervisor		17	2.94
	health mediator/prevention agent		19	3.28
	Cleaner		6	1.04
	Administrative		30	5.18
	Pharmacien		17	2.94
	Laboratory		4	0.69
	Other		12	2.07
Practice type		579		
	Liberal		127	21.93
	Hospital		310	53.54
	Health and prevention centres		81	13.99
	Others		61	10.54
Medical specialization (for physicians)		201		
	General medicine		107	53.23
	Intensive & emergency care		19	9.45
	Specialize medicine		58	28.86
	Surgery		17	8.46
Place of work		579		
	Cayenne area		343	59.24
	Other coastal towns		130	22.45
	Isolated towns of the interior		106	18.31
Year of starting working		579		
	median, IQR		2009	[1997–2017]
Seniority (years)		579		
	median, IQR		12	[4–24]

IQR: inter quartile range.

**Table 2:** History and concerns about COVID-19, by exercise mode.

		Number of Respondents		%	Practice Type				
			*n*	Total	Liberal	Hospital	PPCC	Others	*p*
Total		579							
Personal history of COVID-19		579							
	Yes for sure (positive test)		74	12.78	9.45	13.50	17.28	9.84	0.007
	Yes probably		36	6.22	3.94	8.68	1.23	4.92	
	No		421	72.71	81.10	71.06	64.20	75.41	
	Don’t know		48	8.29	5.51	6.75	17.28	9.84	
Faces severe COVID-19		536							
	Yes, in patients		217	40.49	48.11	41.16	46.25	14.04	0.001
	Yes, among relatives		49	9.14	6.60	8.16	13.75	12.28	
	Both		49	9.14	6.60	10.54	7.50	8.77	
	No		221	41.23	38.68	40.14	32.50	64.91	
At risk of severe COVID-19 (except age)		573							
	Yes		82	14.31	15.75	15.41	7.50	14.75	0.413
	No		470	82.02	81.89	80.66	86.25	83.61	
	Don’t know		21	3.66	2.36	3.93	6.25	1.64	
Worried about the COVID-19 epidemic		579							
	Totally		44	7.60	9.45	6.13	7.41	11.48	0.185
	Rather		308	53.20	48.03	56.13	46.91	57.38	
	Rather not		183	31.61	30.71	30.32	39.51	29.51	
	Not at all		44	7.60	11.81	7.42	6.17	1.64	
Confidence in the management of the epidemic		570							
	Yes fully		18	3.16	5.88	3.00	1.28	1.75	0.008
	Overall		218	38.25	47.90	36.67	30.77	47.37	
	Little		210	36.84	34.45	37.00	42.31	43.86	
	Not at all		108	18.95	11.76	23.33	25.64	7.02	
	Don’t know		16	2.81					

PPCC: Public prevention and care centers, *p*: *p*-value.

**Table 3:** Attitudes towards vaccines in general.

		Number of Respondents		%	Profession			
			*n*	Total	Physicians & Midwives	Nurses	Other	*p*
Total		579	579		244	217	118	
Favourable to vaccinations		547						
	Yes fully		275	50.27	76.76	25.60	37.37	<0.001
	Rather		222	40.59	21.58	63.29	39.39	
	Not		50	9.14	1.66	11.11	23.23	
Unfavourable to certain vaccinations		509						
	Yes		153	30.06	13.04	46.32	39.33	<0.001
	No		356	69.94	86.96	53.68	60.67	
If Yes, vaccine(s) concerned		509						
	Hepatitis B		33	5.70	2.46	10.6	3.39	<0.001
	Human papillomavirus		54	9.33	3.69	14.29	11.86	<0.001
	MMR		10	1.73	1.64	2.30	0.85	0.61
	Yellow Fever		16	2.76	2.05	3.23	3.39	0.67
	Influenza		86	14.85	3.69	26.73	16.10	<0.001
	Rotavirus		25	4.32	6.56	2.76	2.54	0.077
	Meningitis C		15	2.59	2.05	2.76	3.39	0.74
	Pneumococcus		10	1.73	1.64	2.30	0.85	0.61
	Diphtheria-tetanus-polio		10	1.73	1.64	2.30	0.85	0.61
	Shingles		22	3.8	4.51	3.23	3.39	0.75
	Other	Dengue (3), BCG (1), Cholera (1)						
Well informed about vaccinations		576						
	Yes		190	32.99	44.86	26.85	19.66	<0.001
	Almost yes		286	49.65	46.09	53.70	49.57	
	No		100	17.36	9.05	19.44	30.77	
Trust authorities’ information relatives to vaccination		553						
	Yes fully		130	23.51	37.97	11.06	15.74	<0.001
	Rather		298	53.89	54.01	57.21	47.22	
	Rathernot		86	15.55	6.33	21.63	24.07	
	Not at all		39	7.05	1.69	10.10	12.96	
Afraid about adverse effect of vaccinations in general		563						
	Yes fully		76	13.50	3.35	17.37	27.93	<0.001
	Rather		127	22.56	12.55	30.52	28.83	
	Rathernot		246	43.69	54.81	40.38	26.13	
	Not at all		114	20.25	29.29	11.74	17.12	
Agree with the assumption "Vaccines protect others"		564						
	Yes fully		392	69.50	88.43	54.29	57.14	<0.001
	Rather		125	22.16	9.50	33.81	27.68	
	Rathernot		29	5.14	1.24	5.71	12.50	
	Not at all		18	3.19	0.83	6.19	2.68	
Personal vaccinations up to date		579						
	Yes		527	91.02	91.39	94.47	83.90	0.018
	No		27	4.66	5.33	2.30	7.63	
	Don’t know		25	4.32	3.28	3.23	8.47	
Influenza vaccination in 2019–2020		574						
	Yes		183	31.88	52.87	15.74	17.54	<0.001
	No		391	68.12	47.13	84.26	82.46	
Influenza vaccination in 2020–2021		573						
	Yes		140	24.43	42.62	8.84	14.91	<0.001
	No		433	75.57	57.38	91.16	85.09	
Influenza vaccine considered effective		567						
	Yes fully		59	10.41	18.60	3.74	5.41	<0.001
	Rather		272	47.97	62.40	36.92	37.84	
	Rathernot		115	20.28	9.92	28.50	27.03	
	Not at all		42	7.41	2.48	12.62	8.11	
	Don’t know		79	13.93	6.61	18.22	21.62	
Influenza vaccine can have serious adverse effects		568						
	Yes fully		32	5.63	2.07	6.60	11.40	<0.001
	Rather		67	11.80	7.02	15.09	15.79	
	Rathernot		246	43.31	51.24	43.40	26.32	
	Not at all		131	23.06	33.06	17.92	11.40	
	Don’t know		92	16.20	6.61	16.98	35.09	

**Table 4:** Factors associated with willingness to be vaccinated against COVID-19 (Likely / Indecisive / Unlikely or Not). Ordinal multivariate logistic regression.

Willingness to Be Vaccinated against COVID-19														
			Unlikely/Not	Indecisive	Likely/Done		Univariate		Multivariate M1 (*n* = 579)		Multivariate M2 (*n* = 554)		Multivariate M3 (*n* = 527)	
		N	%	%	%	*p*	cOR	95%CI	aOR	95%CI	aOR	95%CI	aOR	95%CI
Total		*n* = 579	(*n* = 141)	(*n* = 65)	(*n* = 373)									
			24.35	11.23	64.42									
Socio-demographic characteristics														
Gender														
	Men	186	11.83	7.53	80.65	<0.001	1		1		1		1	
	Women	393	30.28	12.98	56.74		0.31	[0.21–0.47]	0.42	[0.26–0.68]	0.48	[0.28–0.80]	0.72	[0.37–1.38]
Age (years)														
	18–34	187	37.43	10.7	51.87	<0.001	1		1		1		1	
	35–49	198	22.73	15.15	62.12		1.68	[1.13–2.49]	2.79	[1.71–4.54]	1.8	[1.05–3.07]	1.95	[1.00–3.79]
	50–64	152	15.13	9.21	75.66		3.06	[1.93–4.84]	4.09	[2.36–7.08]	3.01	[1.67–5.45]	3.16	[1.50–6.64]
	65+	42	7.14	2.38	90.48		9.16	[3.15–26.62]	3.38	[1.00–11.47]	2.6	[0.64–10.64]	4.03	[0.55–29.41]
Country of birth														
	France (mainland)	347	17.87	6.92	75.22	<0.001	1		1		1		1	
	French Guiana & West Indies	133	48.87	23.31	27.82		0.17	[0.11–0.25]	0.23	[0.15–0.37]	0.28	[0.16–0.50]	0.52	[0.28–0.99]
	Others countries	99	14.14	10.1	75.76		1.08	[0.64–1.80]	0.7	[0.39–1.25]	0.75	[0.39–1.45]	1.09	[0.46–2.58]
Profession														
	Physicians & midwives	244	9.02	4.1	86.89	<0.001	1		1		1		1	
	Nurses	217	35.48	15.21	49.31		0.15	[0.10–0.24]	0.22	[0.13–0.37]	0.28	[0.16–0.50]	0.5	[0.25–0.99]
	Others	118	35.59	18.64	45.76		0.14	[0.08–0.23]	0.19	[0.11–0.34]	0.22	[0.11–0.42]	0.53	[0.23–1.24]
Exercise mode														
	Liberal	127	21.26	10.24	68.5	0.007	1.3	[0.85–2.01]	0.69	[0.41–1.17]	0.73	[0.40–1.33]	0.61	[0.29–1.24]
	Hospital	310	27.42	9.35	63.23		1		1		1		1	
	Health and prevention centres	81	24.69	22.22	53.09		0.79	[0.49–1.26]	0.91	[0.53–1.56]	0.87	[0.49–1.56]	0.99	[0.48–2.07]
	Other	61	14.75	8.2	77.05		2.02	[1.07–3.81]	1.46	[0.72–2.95]	0.29	[0.68–3.56]	1.33	[0.45–3.90]
Medical specialization (*n* = 201)														
	General medicine	107	9.35	4.67	85.98	0.676	0.46	[0.14–1.46]	/		/		/	
	Intensive & emergency care	19	5.26	0	94.74		1							
	Medical specialty	58	5.17	1.72	93.1		1.32	[0.14–12.55]						
	Surgery	17	11.76	0	88.24		0.54	[0.09–3.22]						
Place of work														
	Cayenne area	343	25.07	9.33	65.6	0.104	0.87	[0.52–1.46]	/		/		/	
	Littoral (others)	130	21.7	18.87	59.43		1							
	Isolated towns of the interior	106	24.62	10	65.38		1	[0.66–1.52]						
Attitudes towards COVID-19														
History of personnal COVID-19														
	Yes	110	22.39	12.37	65.25	0.03	0.74	[0.49–1.14]			/		/	
	No	469	32.73		60.91		1							
Faces severe COVID-19 among patients or relatives (*n* = 544)														
	Yes	315	24.44	12.06	63.49	0.852	1.05	[0.74–1.48]			/		/	
	No	229	26.2	10.92	62.88		1							
At risk of severe COVID-19–19														
	Yes	82	24.39	6.1	69.51	0.323	1.18	[0.72–1.95]			/		/	
	No	491	24.03	11.61	64.36		1							
Worried about COVID-19														
	Yes fully	44	15.91	9.09	75	<0.001	5.6	[2.31–13.6]			5.26	[1.58–17.56]	1.67	[0.24–8.30]
	Rather	308	15.91	10.06	74.03		5.36	[2.87–10.04]			3.99	[1.79–8.90]	2.57	[0.90–7.35]
	Rather not	183	33.88	13.66	52.46		2.06	[1.09–3.88]			1.64	[0.73–3.68]	1.01	[0.35–2.92]
	Not at all	44	52.27	11.36	36.36		1				1		1	
Confidence in the management of the epidemic (*n* = 554)														
	Yes	236	7.63	7.63	84.75	<0.001	3.16	[2.02–4.96]			2.23	[1.34–3.71]	0.76	[0.38–1.50]
	Little	210	23.33	12.38	64.29		1				1		1	
	Not at all	108	60.19	13.89	25.93		0.19	[0.12–0.31]			0.34	[0.20–0.58]	0.61	[0.30–2.24]
Attitudes towards vaccination in general														
Favorable to vaccinations (*n* = 547)														
	Yes fully	275	6.55	2.91	90.55	<0.001	1						1	
	Rather	222	33.33	14.41	52.25		0.12	[0.07–0.19]					0.48	[0.25–0.93]
	Not	50	78	16	6		0.01	[0.00–0.03]					0.25	[0.09–0.71]
Unfavorable to certain vaccinations														
	Yes	153	51.63	15.69	32.68	<0.001	0.11	[0.07–0.16]					...	
	No	356	10.96	6.46	82.58		1							
Confidence in pharmaceutical companies														
	Yes fully	63	1.59	0	98.41	<0.001	6.15	[0.81–46.52]					2.73	[0.33–22.64]
	Rather	243	4.12	4.94	90.95		1						1	
	Rather not	98	39.8	18.37	41.84		0.06	[0.04–0.11]					0.09	[0.04–0.19]
	Not at all	106	74.53	19.81	5.66		0.07	[0.03–0.19]					0.03	[0.01–0.06]
	Don’t know	53	13.21	16.98	69.81		0.23	[0.11–0.48]					0.44	[0.17–1.14]
Well informed about vaccinations														
	Yes	190	18.95	3.68	77.37	<0.001	4.2	[2.56–6.90]					...	
	Rather	286	22.38	12.94	64.69		2.43	[1.58–3.75]						
	No	100	39	21	40		1							
Trust authorities’ informations relatives to vaccination														
	Yes, fully	130	7.69	0.77	91.54	<0.001	70.21	[26.68–184.75]					...	
	Rather	298	17.79	10.07	72.15		17.23	[7.88–37.65]						
	Rathernot	86	45.35	19.77	34.88		3.99	[1.74–9.18]						
	Not at all	39	76.92	15.38	7.69		1							
Afraid about adverses effect of vaccinations in general														
	Yes, fully	76	53.95	23.68	22.37	<0.001	0.07	[0.03–0.13]					...	
	Rather	127	37.01	12.6	50.39		0.16	[0.09–0.30]						
	Rathernot	246	15.45	7.72	76.83		0.54	[0.30–0.99]						
	Not at all	114	9.65	4.39	85.96		1							
Agree with the assomption "Vaccines protect others"														
	Yes, fully	392	13.01	6.63	80.36	<0.001	43.02	[12.24–151.09]					...	
	Rather	125	37.6	20.8	41.6		8.37	[2.35–29.76]						
	Rather not	29	68.97	13.79	17.24		2.44	[0.57–10.40]						
	Not at all	18	83.33	16.67	0		1							
Influenza vaccination in 2019–2020														
	Yes	183	6.56	3.83	89.62	<0.001	7.52	[4.50–12.56]					...	
	No	391	32.74	14.07	53.2		1							
Influenza vaccination in 2020–2021														
	Yes	140	6.43	2.86	90.71	<0.001	7.38	[4.04–13.45]					...	
	No	433	30.02	13.39	56.58		1							
Influenza vaccine considered effective														
	Yes, fully	59	5.08	1.69	93.22	<0.001	20.5	[6.99–60.08]					...	
	Rather	272	9.56	6.25	84.19		7.99	[4.94–12.91]						
	Rather not	115	44.35	14.78	40.87		1							
	Not at all	42	66.67	21.43	11.9		0.34	[0.17–0.69]						
	Don’t know	79	37.97	20.25	41.77		1.16	[0.68–1.98]						
Influenza vaccine can have serious adverse effects														
	Yes, fully	32	59.38	15.62	25	<0.001	0.15	[0.07–0.31]					...	
	Rather	67	37.31	16.42	46.27		0.36	[0.21–0.61]						
	Rather not	246	20.33	7.72	71.95		1							
	Not at all	131	10.69	5.34	83.97		2.09	[1.22–3.59]						
	Don’t know	92	31.52	21.74	46.74		0.4	[0.25–0.64]						

cOR: crude odds ratio, aOR: adjusted odds ratio, 95%CI: confidence interval at 95%, *p*: *p* value, degree of significance.

**Table 5:** Attitudes towards COVID-19 vaccination association with willingness to be vaccinated against COVID-19.

					Willingness to Be Vaccinated against COVID-19			
				%	Unlikely/Not	Indecisive	Likely/Done	
		*n*		Total	%	%	%	*p*
Total		*n* = 579			(*n* = 141)	(*n* = 65)	(*n* = 373)	
					24.35	11.23	64.42	
Attitudes towards COVID-19 vaccination								
Enough informed about COVID-19 vaccine (*n* = 564)								
	Yes, fully		112	19.86	14.29	0.89	84.82	<0.001
	Overall		252	44.68	15.48	7.94	76.59	
	Rather not		123	21.81	30.08	18.70	51.22	
	Not at all		77	13.65	54.55	23.38	22.08	
Confidence in the authorities’ COVID-19 vaccine information (*n* = 541)								
	Totally		77	14.23	1.30	1.30	97.40	<0.001
	Overall		247	45.65	6.07	5.26	88.66	
	Somewhat		126	23.29	38.10	19.05	42.86	
	Not at all		91	26.82	72.53	17.58	9.89	
Confidence in pharmaceutical companies (*n* = 510)								
	Totally		63	12.35	1.59	0.00	98.41	<0.001
	Overall		243	47.64	4.12	4.94	90.95	
	Somewhat		98	19.21	39.80	18.37	41.84	
	Not at all		106	20.78	74.53	19.81	5.66	
Use social network as a source of vaccine information								
	Yes		72	12.43	44.44	15.28	40.28	<0.001
	No		507	87.56	21.50	10.65	67.85	
Think COVID-19 vaccines are effective (*n* = 566)								
	Totally		67	11.83	0.00	1.49	98.51	<0.001
	Overall		256	45.22	6.64	4.30	89.06	
	Don’t know		128	22.61	33.59	20.31	46.09	
	Rather not		71	12.54	54.93	23.94	21.13	
	Not at all		44	7.77	84.09	11.36	4.55	
Worried about RNA vaccine								
	Yes		70	12.09	55.71	20.00	24.29	<0.001
	No		509	87.91	20.04	10.02	69.94	
Worried about DNA vaccine								
	Yes		90	15.54	41.67	14.58	43.75	0.005
	No		509	87.91	22.79	10.92	66.29	
Worried about all type of COVID-19 vaccine								
	Yes		90	15.54	66.67	20.00	13.33	<0.001
	No		509	87.91	16.56	9.61	73.82	
Not worried about a type of COVID-19 vaccine in particular								
	Not worried		166	28.67	18.07	10.84	71.08	0.067
	Worried		413	71.32	26.88	11.38	61.74	
Worried about the serious side effects of these vaccines								
	Totally		89	15.37	66.29	16.85	16.85	<0.001
	Overall		108	18.65	36.11	20.37	43.52	
	Don’t know		47	8.12	29.79	21.28	48.94	
	Somewhat		250	43.18	8.40	4.40	87.20	
	Not at all		69	11.91	4.35	2.90	92.75	
COVID-19 vaccines have more side effects than other vaccines								
	Totally		89	15.37	77.14	14.29	8.57	<0.001
	Overall		108	18.65	44.79	15.62	39.58	
	Don’t know		47	8.12	29.45	19.86	50.68	
	Somewhat		250	43.18	8.67	6.36	84.97	
	Not at all		69	11.92	7.44	1.65	90.91	
Efficacity against severe COVID-19 as a motivation to get the vaccine								
	Yes		303	52.33	13.64	7.34	79.02	<0.001
	No		276	47.67	34.81	15.02	50.17	
Decrease virus transmission as a motivation to get the vaccine								
	Yes		250	43.18	20.13	7.26	72.61	<0.001
	No		329	56.82	28.99	15.58	55.43	
Few or no side effects as a motivation to get the vaccine								
	Yes		205	35.40	32.20	10.73	57.07	0.005
	No		374	64.59	20.05	11.50	68.45	
A greater follow-up as a motivation to get the vaccine								
	Yes		190	32.82	51.05	20.53	28.42	<0.001
	No		389	67.18	11.31	6.68	82.01	
Recommend vaccination to relatives								
	Very likely		124	21.41	1.57	0.79	97.64	<0.001
	Likely		254	43.87	10.48	9.68	79.84	
	Don’t know		63	10.88	28.57	46.03	25.40	
	Unlikely		76	13.13	61.84	25.00	13.16	
	No		62	10.71	95.16	4.84	0.00	
Recommend vaccination to patients								
	Very likely		248	42.83	3.63	1.61	94.76	<0.001
	Likely		144	24.87	13.89	13.19	72.92	
	Don’t know		106	18.31	44.34	32.08	23.58	
	Unlikely		45	7.77	75.56	8.89	15.56	
	No, definitevely		36	6.22	86.11	11.11	2.78	
Number of patients intended to be vaccinated among the last 3 patients (*n* = 344)								
	0 (0%)		150	43.60	31.33	11.33	57.33	<0.001
	1 (33%)		77	22.38	24.68	5.19	70.13	
	2 (66%)		69	20.06	13.04	4.35	82.61	
	3 (100%)		48	13.95	6.25	12.50	81.25	
Arguments for or against COVID-19 vaccination and essential elements for the campaign (recoded)								
	Not enough time since the onset of vaccines		92	15.89	54.35	20.65	25.00	<0.001
			487	84.11	18.69	9.45	71.87	
	Efficacy poorly known		62	10.71	54.84	17.74	27.42	<0.001
			517	89.29	20.70	10.44	68.86	
	Adverses effects poorly known		40	6.91	55.00	20.00	25.00	<0.001
			539	93.09	22.08	10.58	67.35	
	Understandable information for the population		144	24.87	25.69	8.33	65.97	0.440
			435	75.13	23.91	12.18	63.91	
	Cultural issues, borders		81	13.99	9.88	3.70	86.42	<0.001
			498	86.01	26.71	12.45	60.84	
	Logistics issues		73	12.61	10.96	8.22	80.82	0.006
			506	87.40	26.28	11.66	62.06	
	Many comorbidities in French Guiana		71	12.26	14.08	11.27	74.65	0.090
			508	87.74	14.08	11.27	74.65	
	Efficacy on Gamma variant not known		47	8.12	38.30	14.89	46.81	0.028
			532	91.88	23.12	10.90	65.98	
	Precariousness		43	7.43	13.95	13.95	72.09	0.247
			536	92.57	25.19	11.01	63.81	
	Inadequacy of the health system		17	2.93	17.65	5.88	76.47	0.559
			562	97.07	24.56	11.39	64.06	

*p*: *p*-value.

## Data Availability

All the relevant data for our analyses are fully described in the paper and can be made available on request. All data used for the analysis are available on request from the corresponding author.
